# Editorial: Induced plant resistance against pathogens by application of bioactive molecules

**DOI:** 10.3389/fpls.2023.1282909

**Published:** 2023-09-20

**Authors:** Feng Zhu, Jing Shang, Sek-Man Wong

**Affiliations:** ^1^ College of Plant Protection, Joint International Research Laboratory of Agriculture and Agri-Product Safety, the Ministry of Education of China, Yangzhou University, Yangzhou, Jiangsu, China; ^2^ College of Agronomy, Sichuan Agricultural University, Chengdu, Sichuan, China; ^3^ Department of Biological Sciences, National University of Singapore, Singapore, Singapore; ^4^ National University of Singapore Suzhou Research Institute, Suzhou Industrial Park, Suzhou, Jiangsu, China

**Keywords:** induced plant immunity, PTI, ETI, SAR, RNAi, reactive oxygen species, plant hormones, bioactive molecules

Plants constantly face many kinds of pathogens, such as bacteria, oomycetes, fungi, viruses, and nematodes. Plant diseases caused by these pathogens may result in huge crop yields and quality losses (Savary et al., 2019; Zhu et al., 2023a). Currently, the application of conventional agrochemicals remains the main approach to control crop diseases in agricultural production. However, the ever-increasing environmental and social problems of agrochemicals such as pathogens resistance, destruction of ecological balance and biodiversity, and environmental pollution mean that ecologically safer and more sustainable technologies are urgently needed.

During the co-evolution between plants and pathogens, plants have evolved complex defense mechanisms and strategies against various pathogens infection. For example, PAMP-triggered immunity (PTI), effector-triggered immunity (ETI), systemic acquired resistance (SAR) and RNA interference (RNAi) are the major plant immune defense systems to protect the host from various pathogens attacks (Ngou et al., 2021; Yuan et al., 2021; Chang et al., 2022; Zhu et al., 2023b). Several studies indicated that the induction of plant immunity can be triggered by exogenous bioactive molecules, such as proteins, peptides, phytohormones, nanoparticles, oligosaccharides, microbial biocontrol agents, and so on. This has raised the prospect that induced plant resistance by application of bioactive molecules could serve as a sustainable approach in crop protection.

This Research Topic combines 6 publications, including 3 review articles and 3 research articles, covering several aspect of induced plant resistance. The featured in-depth topic reviews and research articles provide readers a convenient way to expand and understand the current knowledge and status of the induced plant resistance.

Exogenous application of inorganic salts significantly enhance resistance against various pathogens in multiple plant species (Deliopoulos et al., 2010; Zhu et al., 2023b). For example, application of K_2_HPO_4_ or KH_2_PO_4_ triggered the activities of defense-related enzymes in bean plants and increased resistance to *Uromyces appendiculatus* (Devi et al., 2020). Sun et al. have investigated the effects of exogenous application of sodium silicate (Na_2_SiO_3_·9H_2_O) on the occurrence of *Fusarium wilt* in cucumber and its mechanisms. They have provided evidence that exogenously applied silicon enhances resistance to cucumber wilt disease by up-regulating the antioxidant system, photosynthetic capacity, and stomatal opening in cucumber leaves.

The use of biological control agents for the management of plant pathogens is an ecologically safer and sustainable strategy in agriculture production (Köhl et al., 2019). Several biocontrol bacteria exhibit strong antimicrobial activities because they can produce various antimicrobial substances and induce host resistance against various plant pathogens. Yang et al. have isolated a potential biocontrol bacterium (*Burkholderia gladioli* strain KJ-34) from the rhizosphere soil of rice. They reveal that *B. gladioli* strain KJ-34 shows broad-spectrum strong antifungal activities against multiple fungal pathogens and produces various biological control metabolites such as benzoylstaurosporine, morellin and scopolamine. The KJ-34 not only inhibits mycelial growth and spore germination of multiple filamentous fungi but also induces resistance to *Botrytis cinerea* and *Magnaporthe oryzae* in tomato and rice, respectively.

Reactive oxygen species (ROS) and plant hormones, such as jasmonic acid (JA), salicylic acid (SA), and ethylene (ET) play important roles in plant immunity and resistance (Berens et al., 2017; Mittler et al., 2022). Su and Gassmann review how the host controls chloroplast ROS (cROS) accumulation during ETI. They summarize possible cytoplasmic regulatory mechanisms of cROS accumulation during ETI from three aspects, such as, selective mRNA decay, translational regulation, and autophagy-dependent formation of Rubisco-containing bodies (RCBs). Kesel et al. have provided an interesting finding that foliar diproline (cyclo(L-Pro-L-Pro)) treatment induces resistance to root-knot nematode *Meloidogyne graminicola* (*Mg*) in the same and subsequent rice generations. Furthermore, diproline-induced resistance against *Mg* in rice is associated with disturbances in the iron (Fe) homeostasis, ET and nitric oxide (NO) pathways.

Various kinds of oligosaccharides, such as chitosan, chitin, and β-glucans, function as PAMPs or DAMPs and consequently activate plant immunity (Guarnizo et al., 2020; Zhu et al., 2023b). Chitosan or its fragments have been shown to induce plant immunity against fungal pathogens in various crops. Mukarram et al. review the current knowledge of chitosan’s antimicrobial and insecticidal potential. Chitosan supports plant growth and development and protects against microbial entities such as fungi, bacteria, viruses, nematodes, and insects. They also summarize the action mechanism of chitosan-induced resistance against various pathogens and insects. The chitosan perception is proved to have crosstalk with several other signaling pathways, such as phytohormones (SA, indole acetic acid (IAA), gibberellic acid (GA), abscisic acid (ABA), JA), ROS and antioxidant metabolism, and cellular signaling molecules via Ca^2+^, NO, transcription factors (TFs), and defensive gene activation.

Pathogenesis-related proteins (PRs) are small proteins that play important roles in plant defense responses against biotic stresses (Kattupalli et al., 2021). Application of multiple bioactive molecules enhance host resistance against various pathogens through inducing the accumulation of PRs. Currently, PRs are divided into 17 families (PR-1 to PR-17) based on their physicochemical properties, such as defensins and chitinases. Lopes et al. comprehensively review the mechanisms of action of PR-10 in the plant’s defense response against biotic stresses (viruses, bacteria, fungi, oomycetes, nematodes and insects). They summarize the modes of action of PR-10 crosstalk with TFs, phytohormones pathways, physical interactions with effector proteins or pattern recognition receptors and other signaling molecules involved with the plant’s defense system.

We hope that this Research Topic helps readers to increase understanding of the molecular mechanisms and their potential applications in the activation of plant defense by bioactive molecules. A deeper understanding of the molecular mechanisms and their potential applications in the activation of plant defense by bioactive molecules will be crucial to developing effective and sustainable tools to control plant diseases in crop protection.

## Author contributions

FZ: Funding acquisition, Writing – original draft, Writing – review & editing. JS: Writing – review & editing. SW: Writing – review & editing.
